# Cellular Senescence in Cardiovascular Diseases: From Pathogenesis to Therapeutic Challenges

**DOI:** 10.3390/jcdd10100439

**Published:** 2023-10-23

**Authors:** Dan Li, Yongnan Li, Hong Ding, Yuqin Wang, Yafei Xie, Xiaowei Zhang

**Affiliations:** 1 Department of Cardiovascular Medicine, Lanzhou University Second Hospital, Lanzhou 730030, China; lid2021@lzu.edu.cn (D.L.); dingh0110@163.com (H.D.); wangyq21@lzu.edu.cn (Y.W.); xieyf0226@163.com (Y.X.); 2 Department of Cardiac Surgery, Lanzhou University Second Hospital, Lanzhou 730030, China; lyngyq2006@foxmail.com

**Keywords:** cellular senescence, cardiovascular disease, SASP, senolytics, senomorphics

## Abstract

Cellular senescence (CS), classically considered a stable cell cycle withdrawal, is hallmarked by a progressive decrease in cell growth, differentiation, and biological activities. Senescent cells (SNCs) display a complicated senescence-associated secretory phenotype (SASP), encompassing a variety of pro-inflammatory factors that exert influence on the biology of both the cell and surrounding tissue. Among global mortality causes, cardiovascular diseases (CVDs) stand out, significantly impacting the living quality and functional abilities of patients. Recent data suggest the accumulation of SNCs in aged or diseased cardiovascular systems, suggesting their potential role in impairing cardiovascular function. CS operates as a double-edged sword: while it can stimulate the restoration of organs under physiological conditions, it can also participate in organ and tissue dysfunction and pave the way for multiple chronic diseases under pathological states. This review explores the mechanisms that underlie CS and delves into the distinctive features that characterize SNCs. Furthermore, we describe the involvement of SNCs in the progression of CVDs. Finally, the study provides a summary of emerging interventions that either promote or suppress senescence and discusses their therapeutic potential in CVDs.

## 1. Introduction

CS entails cycle arrest and concurrent changes in various metabolic pathways, leading to morphological and functional alteration [1]. Under physiological conditions, CS facilitates cellular turnover and organ repair, forming a closely regulated process influenced by many kinds of cells. Nevertheless, SNCs often evade timely elimination and replacement within aging tissues or pathological conditions, resulting in their accumulation and subsequent organ dysfunction [2].

**Figure 1:**
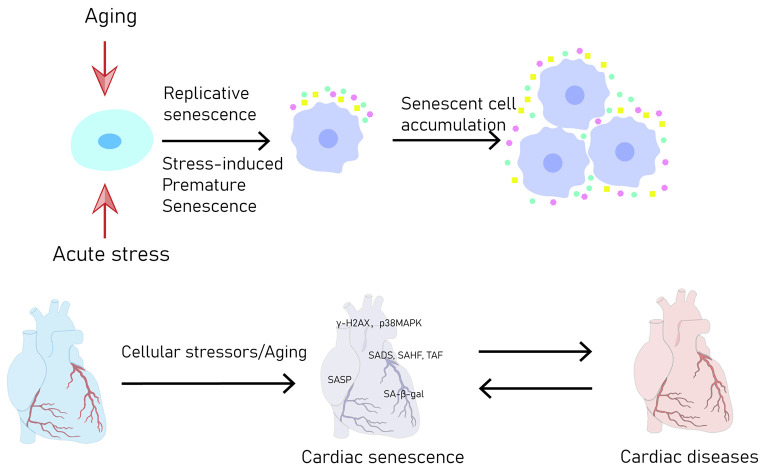
Accumulation of SNCs leads to CVDs. γH2AX, phosphorylated histone H2AX; p38MAPK, p38 mitogen-activated protein kinase; SADS, senescence-associated distention of satellites; SAHF, senescence-associated heterochromatin foci; TAF, telomere-associated foci; SASP, senescence-associated secretory phenotype; SA-β-gal, senescence-associated β-galactosidase.

Among multiple chronic fatal diseases, CVDs are the most prevalent, emerging as a significant factor in elderly mortality rates, especially as global demographics increasingly lean toward older age groups [3]. Across a range of cardiovascular conditions, both ischemic and non-ischemic cardiomyopathies, clinical data have revealed a significant relationship between cellular senescence and unfavorable cardiac outcomes [2]. Nevertheless, the exact function of SNCs in these conditions remains enigmatic, as certain instances exhibit both harmful and advantageous effects [4,5,6,7,8,9]. Therefore, although the multiple causes of CVDs have been elucidated due to advances in genomics, epigenomics, proteomics, and single-cell analysis, in this review we prioritize the role of CS in CVDs (Figure 1). Regulatory signals and possible points of intervention in different types of cardiovascular SNCs are reviewed. Furthermore, it outlines various promising targets for manipulating the process of CS. The ultimate goal is to offer a fresh perspective for the development of compounds that regulate senescence and enhance the clinical transformation of cardioprotective lead compounds.

## 2. Definition and Mechanisms of CS

**Figure 2:**
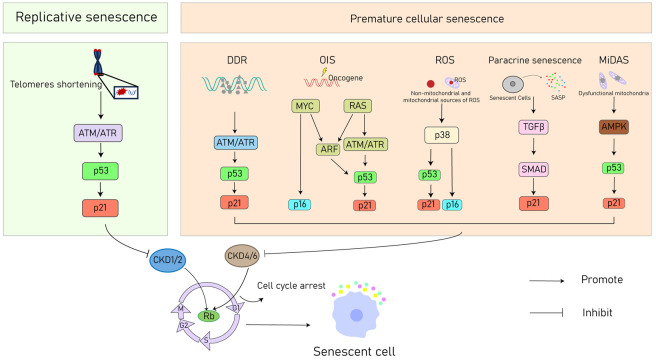
Overview of molecular mechanisms leading to CS. ATM/ATR, ataxia–telangiectasia mutated or ataxia–telangiectasia Rad3-related; DDR, DNA damage response; OIS, oncogene-induced senescence; ROS, reactive oxygen species; SASP, senescence-associated secretory phenotype; TGF-β, transforming growth factor-β; SMAD, small mother against decapentaplegic; AMPK, adenosine 5′-monophosphate (AMP)-activated protein kinase; Rb, retinoblastoma protein.

Although knowledge about the role of CS in health and disease is controversial, its basic definitions and principles still exhibit a certain degree of consensus. In a historical context, senescence was first defined by Hayflick over 70 years ago. His discovery revolved around the observation that human diploid fibroblasts exhibited a limited ability to divide, primarily attributed to telomere shortening [10]. However, senescence can be triggered even when there is no observable telomere loss or dysfunction, in various circumstances. This specific form of senescence has been categorized as ‘premature’ due to its occurrence prior to the stage when it is initiated by the shortening of telomeres [11]. Unlike the former, this variant is not linked to ongoing telomere shortening, but rather to non-telomeric DNA damage and sustained mitogenic stimulation, a form known as stress-induced premature senescence (SIPS) [12]. Fundamentally, CS embodies a cellular state marked by a durable halt in proliferation as a response to diverse stressors, frequently accompanied by the generation of a related secretome recognized as the SASP [13]. In this section, the discussion will briefly cover the involvement of cellular stressors in triggering CS, with particular emphasis on factors such as telomere shortening, oxidative-related senescence, DNA damage, chemotherapy, oncogene activation, mitochondrial dysfunction, epigenetics, the SASP process, and other factors (Figure 2).

### 2.1. Replicative CS

In line with Hayflick’s hypothesis, contemporary knowledge suggests that, as human cells are cultured over time, telomeres experience gradual shortening, culminating in cells reaching their “Hayflick limit”. This occurrence is denoted as replicative senescence (RS), given that it emerges as a consequence of replication [10]. Telomeres, situated at the termini of linear chromosomes, serve as protective structures. They are composed of repetitions of the tandem sequence TTAGGG and are linked to the shelterin complex [10]. Such an arrangement acts as a protective barrier against chromosomal damage, as it prevents fusion between chromosomes and degradation, thereby preserving genome integrity [14]. With each round of replication, there is a decrease in the number of telomeres repeating, as DNA polymerase cannot fully replicate the lagging strands. One of the most extensively characterized mechanisms in RS is the notable reduction in telomere length that occurs with each successive cellular proliferation [10].

The gradual shortening of telomeres results in the uncovering of DNA termini, ultimately triggering a DNA damage response (DDR). This reaction results in the occurrence of chromosomal unsteadiness and the formation of atypical connections between chromosomes, along with the triggering of inhibitors that regulate cell cycle progression such as p53, p21^CIP1^, WAF1, or p16^INK4a^/retinoblastoma protein (Rb). These inhibitors induce cell cycle halt at the G1 phase [15]. The fact that RS depends on telomere shortening becomes clear when it is overcome by the ectopic expression of the catalytic subunit of the human telomerase reverse transcriptase (hTERT) holoenzyme, which elongates telomeres and lessens the impact of the end replication problem [16]. The constrained longevity of the majority of primary human cells is linked to the absence of telomerase expression in human somatic cells, unlike stem cells. Consequently, human somatic cells cannot sustain telomeres at a length that is adequate to suppress DDR [17]. In conclusion, the key molecular events underlying RS seem to encompass telomere shortening and the repression of telomerase during the process of biological aging [11].

### 2.2. Premature CS

#### 2.2.1. DNA Damage-Induced Senescence

Among the heart and blood vessels, the genome is uninterruptedly exposed to diverse internal and external factors, encompassing ischemia, lipids, blood flow, and aging. These factors inevitably trigger genome instability and transcription disorders [2]. The senescent phenotype can be induced by any stressor that results in persistent DNA damage [18]. DNA damage refers to modifications made to the DNA structure, which can result in changes to its coding properties or its capability of involvement in transcription or replication [19]. Various forms of DNA damage have been identified, including single-strand breaks (SSBs), double-strand breaks (DSBs), DNA–protein cross-links, and insertion or deletion mismatches [20]. Among these, the DNA damage type most consistently associated with aging are DSBs [21].

The DDR is defined by the activation of sensor kinases (ataxia–telangiectasia mutated [ATM] or ataxia–telangiectasia Rad3-related [ATR]), the accumulation of DNA damage foci that contain phosphorylated histone H2AX (γH2AX), and the engagement of checkpoint proteins like p53 and the CDK inhibitor p21 (CDKN1A). Eventually, this cascade culminates in the halting of the cell cycle, resulting in senescence [22]. As a result, DNA damage foci exhibiting positive γ-H2AX staining have been validated as dependable indicators of sustained DNA damage and senescence [23]. Mechanistically, DNA damage and senescence establish a feedback loop. Senescence can disrupt cytoplasmic DNA clearance, activating the senescence-sensing mechanism through cyclic GMP–AMP synthase (cGAS) and triggering the stimulator of interferon genes (STING). This activation promotes senescence and the secretion of SASP [24].

Furthermore, telomere shortening represents a form of DNA damage. Consequently, the existence of DNA damage foci within the telomere region acts as a distinguishing feature of CS referred to as telomere-associated foci (TAF). Detecting TAF at telomeres offers solid proof for quantifying telomere damage as a response to stress. Co-localization studies, often utilized to confirm the existence of TAF, encompass the examination of DDR factors (such as p53-binding protein 1 (p53BP1) and γ-H2AX) as well as telomere repeats [25]. The targeted induction of TAF using the TRF1–Fok1 fusion protein triggers CS in cardiomyocytes, without regard to the length of telomere. These findings indicate that ongoing DNA damage at telomeres could initiate CS in cardiomyocytes [26].

#### 2.2.2. Oncogene-Induced Senescence

In 1997, it was discovered that, in early passage normal human and murine fibroblasts, oncogenic Ras leads to an initial phase of hyperproliferation, along with subsequent essentially irreversible growth arrest. This arrest closely resembles the phenotypic characteristics of RS. This phenomenon has since been referred to as oncogene-induced senescence (OIS) [27]. The activation of oncogenes initiates a hyper-replicative phase, subsequent to the activation of the DNA damage checkpoint response. This phase leads to an elevation in the quantity of active replicons and alterations in the progression of DNA replication forks [28]. In contrast to RS, OIS is not preventable by the expression of hTERT, underscoring its dissociation from telomere erosion [29]. The involvement of the p53 and p16^INK4A^-RB pathways is a common feature observed in cells experiencing both RS and OIS, albeit within specific contexts [30]. Multiple studies have suggested that CS can impede tumor formation by inducing a prolonged cell cycle withdrawal and restricting the generation of cancer stem cells [31,32,33,34].

#### 2.2.3. Oxidative Stress-Induced Senescence

The hypothesis of the free radical theory of aging, advanced earliest by Denham Harman, posits that an excess of ROS causes harm to macromolecules. The gradual accumulation of this macromolecular damage subsequently results in cellular and organ dysfunction as time progresses [35]. Senescence can be induced by oxidative stress through direct effects on telomeres, irrespective of their length, or by impacting genomic or mitochondrial DNA (mtDNA) [36]. ROS production can be induced by both exogenous and endogenous stressors, via either mitochondrial or non-mitochondrial sources [37]. ROS can play a signaling role in both the induction and maintenance of senescence by directly causing oxidative modifications in subcellular structures, such as nucleic acids and enzymes. This oxidation, in turn, triggers the activation of oncogenes, DNA damage, and reduced telomerase activity [38]. If the presence of stress is persistent and surpasses the antioxidant capacity to counteract it, the process of senescence persists. Alternatively, when antioxidant defenses are sufficient, the senescence process transitions towards autophagy, restoring a proliferative state while reducing the expression of senescence markers [39]. Consequently, oxidative stress stands as one of the essential and pivotal mechanisms contributing to stressor-induced CS.

#### 2.2.4. Paracrine Senescence

Within the SASP, there are three primary groups of secretory proteins: soluble signaling factors, proteases, and insoluble proteins or extracellular membrane (ECM) constituents. The secreted elements originating from SASP influence the nearby tissue microenvironment surrounding SNCs, thereby causing phenotypic changes in neighboring non-SNCs. These molecules and pathways associated with SASP critically mediate the pathophysiological impact of SNCs in tissue damage, chronic inflammation, and tumorigenesis. This is accomplished through intercellular communication and the creation of distinct microenvironmental conditions [40]. One advantageous role of SASP is its capacity to recruit immune cells, promote tissue homeostasis, initiate tissue repair and remodeling via the elimination of dysfunctional cells, and impede tumor growth by preventing the transmission of genetic alterations to future cell generations [41]. Nonetheless, in addition to the advantages, SASP can also exert detrimental effects. It can stimulate a pro-inflammatory microenvironment, perpetuate chronic inflammation, and facilitate tumor progression. In terms of mechanisms, SASP serves as a two-sided coin, generating both advantageous and disadvantageous effects on human diseases [41].

#### 2.2.5. Mitochondrial Dysfunction-Associated Senescence (MiDAS)

Maintaining normal mitochondrial and cardiac function requires mitochondrial fusion, while fission supports the elimination of dysfunctional, depolarized mitochondria through the mechanism of mitophagy [42,43]. A disruption in the balance between mitochondrial fission and fusion that causes an excess of mitochondrial fusion, similar to what is seen in senescence, brings about the retention of impaired mitochondria and the build-up of oxidized proteins. This aggregation could potentially worsen the senescent phenotype [43]. Mitochondrial dysfunction is strongly correlated with an augmented generation of ROS, resulting in heightened oxidative damage. This damage encompasses sulfhydryl oxidation, lipid peroxidation, and mutations in mtDNA [44,45]. Metabolic dysfunction and irregular mitochondrial dynamics are particularly essential in facilitating the senescence of cardiomyocytes.

#### 2.2.6. Epigenetically Induced Senescence

Epigenetic modifications involve DNA methylation, histone acetylation, chromatin remodeling, and non-coding RNAs [46]. SNCs harbor extensive alterations in DNA methylation compared to proliferating cells. These changes include global hypomethylation and hypermethylation of CpG islands [47]. Histone modification directly modifies chromatin structure and attracts adaptor or effector proteins with binding domains that aid in chromatin remodeling [46]. In addition to their involvement in senescence and the progression of CVDs, non-coding RNAs, including miRNAs and lncRNAs, contribute to regulatory processes [48,49,50].

#### 2.2.7. Other Factors

In addition to the mechanisms described above, new studies propose that CS can result from other factors. As an illustration, according to aging theory, excessive activation of nutrient sensing pathways, such as targets of insulin/insulin-like growth factor 1 (IGF-1) signaling and rapamycin, is linked to accelerated aging and a reduced lifespan [51]. Consequently, pursuing moderate dietary restriction, inducing genetic mutations, or employing chemical agents to lower the activity of nutrition-sensitive signaling pathways can serve to extend the lifespan of organisms [52]. Additionally, the dysregulation of systemic humoral pathways has been closely linked to CS, including cases involving a deficiency of Klotho [53]. Finally, protein folding is implicated in CS processes, with ER stress being involved in initiating or sustaining aging phenotypes [54].

## 3. Hallmarks of CS

**Figure 3:**
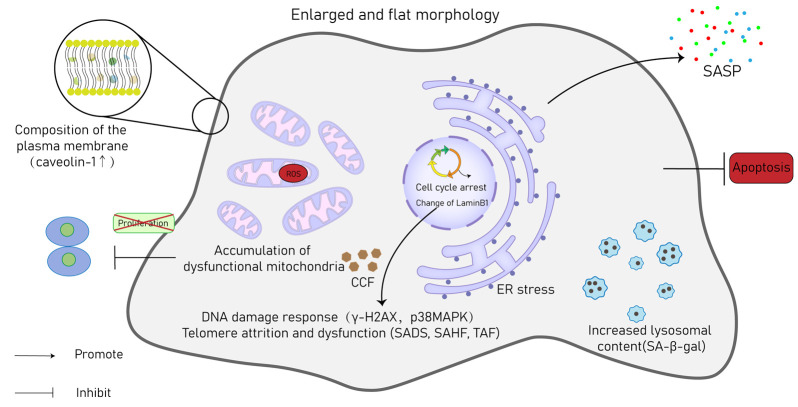
Hallmarks of SNCs. γH2AX, phosphorylated histone H2AX; p38MAPK, p38 mitogen-activated protein kinase; SADS, senescence-associated distention of satellites; SAHF, senescence-associated heterochromatin foci; TAF, telomere-associated foci; ER, endoplasmic reticulum; CCF, cytoplasmic chromatin fragment; SA-β-gal, senescence-associated β-galactosidase; SASP, senescence-associated secretory phenotype.

Various conditions trigger CS, and SNCs exhibit several distinguishing characteristics that enable their detection in both in vitro and in vivo settings (Figure 3). In the heart and other tissues, specific hallmark characteristics are indicative of SNCs, serving as indirect evidence of the onset of senescence.

A prominent characteristic of CS is the distinct morphological alterations that transpire as it advances. Ball observed that senescent fetal cardiomyocytes consistently display an enlarged and vacuolized cell body [55]. Various senescent cardiovascular cells also show a flattened morphology, an enlarged volume, and the presence of vacuoles [56,57,58]. Consequently, these morphological changes could serve as markers for identifying SNCs in CVDs, even in the absence of cell-type specificity. The most prevalent modification in the plasma membrane composition of SNCs is the heightened expression of caveolin-1, which was consistently observed [59]. An increased number of mitochondria is evident in SNCs, relative to non-SNCs [60]. Even though there is an increased quantity of mitochondria in SNCs, their membrane potential is reduced, leading to the liberation of mitochondrial enzymes such as EndoG and an augmented production of ROS [61]. Mitochondrial dysfunction is considered both a marker and an inducer of stem cell aging [62]. The diminished mitophagy brings about the gathering of aged and impaired mitochondria, contributing primarily to the increased mitochondrial content [63]. SNCs exhibit elevated activity of senescence-associated β-galactosidase (SA-β-gal) due to heightened lysosomal density. Therefore, SA-β-gal is widely used as a prominent hallmark for identifying SNCs [64]. Sudan Black B (SBB) is an alternative marker that can be employed to detect the accumulation of lysosomes. It provides another means of identifying the presence and abundance of lysosomes in cells [65]. Although the elevated activity of lysosomes, indicated by elevated SA-β-gal expression, is commonly linked to SNCs, it is not a definitive hallmark of CS, since the constitutive expression of SA-β-gal has also been observed in non-SNCs [66]. The emergence of cytoplasmic chromatin fragments (CCFs) is a common characteristic observed in SNCs, which is accompanied by the depletion of lamin B1, an essential nuclear structural protein [67]. These CCFs have the ability to be released into the external environment through exosomes and trigger DDR in other cells [68].

As previously described, most stressors that lead to CS activate either the p53/p21 or p16^Ink4a^/retinoblastoma protein pathways. The buildup of cell cycle-suppressing proteins, including p16 (p16^INK4A^), p53, or p21, is frequently employed as a hallmark of senescence [69]. Alongside p53, p21, and p16^Ink4a^, other widely recognized markers of CS encompass elevated levels of the p38 mitogen-activated protein kinase (p38MAPK) or γ-H2AX, which reflect the activation of DDRs. Senescence-associated heterochromatin foci (SAHF) and senescence-associated distention of satellites (SADs) are also recognized as markers of SNCs [70,71].

Telomere-related DNA damage, referred to as telomere dysfunction-induced foci (TAF), is identified through the co-localization of γ-H2AX and p53-binding protein 1 (p53BP1) with telomeres. In fibroblasts, exposure to irradiation or application of hydrogen peroxide (H_2_O_2_) results in an elevated formation of TAF. This phenomenon has also been noted to increase in the liver, gut, and heart as individuals age [26,72]. SNCs cease to divide. A senescent phenotype is indicated by the reduction in proliferation markers, like Ki67, along with the lack of incorporation of 5-bromodeoxyuridine (BrdU) or ethynyl deoxyuridine (EdU) [30]. The SASP encompasses a multitude of proteins, which are secreted into the extracellular milieu by SNCs. Although SASP is critically involved in the pathophysiological behavior of SNCs, its nonspecific and heterogeneous character hinders its application as an unequivocal marker for senescence [73]. Nevertheless, evaluating the composition of SASP quantitatively could serve as a means to differentiate between different senescence programs [74].

The characterization of cells as senescent is a multifaceted process. As there is no single specific biomarker capable of definitively identifying SNCs, a combination of markers is typically employed to confirm the presence of a senescent cellular phenotype.

## 4. Senescence in Specific Cardiac Cell Types

The impact of senescence on cells is highly influenced by their cell type, tissue composition, and organ context, leading to considerable variation in its effects. Different cell types make up the intricate structure of the heart, including cardiomyocytes, endothelial cells, fibroblasts, vascular smooth muscle cells (VSMCs), immune cells, and cardiac progenitor cells (CPCSs) [75]. Extensive in vitro and in vivo studies have demonstrated that all types of cardiovascular cells, both during the natural aging process and in the context of CVDs, can undergo senescence [1]. Hence, the function of CS in age-related CVDs can arise from alterations in multiple cell types (Table 1).

### 4.1. Cardiomyocytes

CS has traditionally been recognized as an irreversible G1 arrest state in mitotic cells. Nevertheless, in the heart, a significant proportion of cardiomyocytes differentiate into terminally differentiated cells that cease to undergo cell division immediately after birth. Despite being mostly non-dividing, cardiomyocytes from aged rats and mice exhibit telomere shortening, similar to normal human heart tissue [10]. Despite enduring debate that span decades, an increasing body of research indicates that cardiomyocytes possess mechanisms that can trigger senescence [11].

The manifestation of impaired contractility and disrupted conduction patterns in senescent cardiomyocytes can give rise to cardiomyopathies or arrhythmias. As a case in point, in a mouse model of Duchenne muscular dystrophy (DMD), the absence of dystrophin in cardiomyocytes resulted in the emergence of a senescent phenotype. Moreover, mutations in lamin A (LMNA) are linked to dilated cardiomyopathy and this may be linked to genomic instability that occurs as a result of nuclear membrane disruption. This disruption results in increased regions of open chromatin [76]. Compelling evidence indicates that CS is critically involved in mediating cardiomyopathy induced by chemotherapy and radiotherapy, such as with anthracyclines and doxorubicin.

### 4.2. Endothelial Cells

By releasing vasoactive compounds and growth factors, endothelial cells have a crucial function in regulating vascular tone and vasodilation [77]. The continuous exposure of these cells to various stressors renders them susceptible to damage, which has the potential to induce senescence. Therefore, well-vascularized tissues, especially those with a high density of endothelial cells, tend to bear a greater load of SNCs [78]. The heightened secretion of endothelin-1 (ET-1) and reduced production of nitric oxide by senescent endothelial cells create a substantial interplay between these SNCs and the surrounding cardiac cell populations [78]. Consequently, vascular inflammation and impaired vasodilation ensue because of this phenomenon, establishing a self-amplifying cycle where the amassment of senescent endothelial cells leads to vascular disorder, and subsequently also the other way around [79]. Endothelial cell senescence results in compromised vasodilation and vascular dysfunction, contributing to conditions like atherosclerosis, heart failure (HF) with preserved ejection fraction (HFpEF), or pulmonary hypertension (PH) [80].

### 4.3. Cardiac Fibroblasts

Cardiac fibroblasts are crucial in regulating ECM remodeling, reorganization, and paracrine communication within the cardiac microenvironment [81]. To aid in adhesion, SNCs express integrins and matrix metalloproteinases (MMPs) that help preserve ECM structure and integrity [82]. Following an acute myocardial infarction (MI), the p53 and p21 pathways are upregulated, culminating in the senescence of cardiac fibroblasts [83]. Inducing senescence in cardiac fibroblasts can have positive and negative impacts—advantageous in the context of chronic wound healing after MI, yet potentially harmful during myocardial fibrosis associated with aging [76]. Cardiac fibroblasts also engage in paracrine signaling to modulate processes like proliferation, hypertrophic growth, and cardiomyocyte senescence [81]. The transition of activated cardiac fibroblasts from a pro-inflammatory condition to an anti-inflammatory condition is crucial in the formation of scar tissue following an acute MI [84]. Navitoclax, a senolytic agent administered systemically, has been found to improve outcomes following ischemia-reperfusion injury by clearing SNCs. Thus, it is essential to strike a balance between the beneficial effects of fibroblast senescence and the potentially detrimental consequences of senescence [85].

### 4.4. VSMCs

VSMCs collaborate with endothelial cells to regulate critical aspects of vascular function, including arterial pressure regulation, vascular tone maintenance, and blood flow coordination [86]. CS can be triggered in VSMCs through various mechanisms, like telomeric shortening, DNA damage, oxidative stress, and dysfunction in autophagy [87]. Elevated SA-β-gal activity and dysregulated transcriptome involving p16, p21, and Rb proteins are observed in senescent VSMCs. Additionally, they show increased expression of inflammatory cytokines [88]. VSMCs senescence contributes to vascular diseases like atherosclerosis and pulmonary hypertension [89]. The secretion of monocyte chemoattractant protein-1 (MCP-1) and macrophage inflammatory protein-1α/β (MIP1α/β) stimulates the engagement of monocytes, macrophages, and lymphocytes, consequently promoting plaque growth and aggravating the likelihood of rupture [90]. Moreover, the CS of VSMCs may also play a role in the pathophysiology of PH through SASP [91].

### 4.5. Immune Cells

Immune cells, particularly macrophages and T cells with senescent-like characteristics, have gained significant attention in recent studies for their substantial roles in CVDs [76]. The susceptibility of atherosclerotic plaques to rupture is heightened in cases where there is a higher abundance of macrophages, as elucidated by a recent study [92,93,94]. Notably, in the atrioventricular node, cardiac macrophages have been shown to enhance electrical conduction. In the context of CVD, leukocytes exhibiting short telomeres, which are a characteristic feature of senescence, have been identified within atherosclerotic coronary arteries [95]. A positive feedback loop can be established through the secretion of inflammatory cytokines, chemokines, and metalloproteinases by senescent foamed macrophages exhibiting senescence markers within atherosclerotic plaques [96]. Apart from macrophages, senescent-like T cells can also contribute to the development of chronic inflammatory diseases, encompassing CVDs [97] such as atherosclerotic disease, hypertension, coronary artery disease (CAD), acute coronary syndromes, and HF [98,99,100,101].

### 4.6. Cardiac Progenitor Cells

CPCs are a unique type of progenitor cell that can differentiate into cardiac myocytes and endothelial cells. The lifespan of these cells is influenced by telomerase activity and telomere length, like other cell types [102]. As humans age, CPCs in the heart tend to become senescent, which can lead to an inability to sustain homeostasis, repair damaged tissue, and regenerate after injury [103]. Various research groups have investigated the use of senolytic medications to enhance CPC function, indicating that CPC senescence could be a viable target for future interventions targeting CVDs [104].

Communication between cells in the myocardial microenvironment is significant in regulating cardiac homeostasis and the aging process. Non-myocyte cells can modulate the physiological functions and senescence of cardiomyocytes. At the same time, cardiomyocytes also exert their influence on non-myocytes, partially through paracrine signaling [105].

## 5. Physiology and Pathophysiology of CS in the Cardiovascular System

### 5.1. Senescence in Heart Development

Numerous investigations have explored the involvement of CS in cardiac embryonic development and the remodeling of cardiac tissues. Senescence assumes a crucial role during embryogenesis, often referred to as developmental senescence, wherein the elimination of SNCs holds significance for morphogenesis [5]. Both SNCs and numerous standard components of the SASP are discernible in the developing embryos of experimental animals [106]. Additional support stems from observations highlighting that abnormalities in senescence-related proteins throughout the developmental process are connected to birth defects of the heart [107,108]. Hence, it becomes evident that senescence actively participates in the process of embryonic development of the four-chambered mammalian heart, whereby aberrant senescence signaling is linked to various congenital heart defects in this context [1].

While CS indeed contributes to organismal remodeling and wound healing, compelling data strongly indicate that persistent senescence in the process of aging is also intertwined with a variety of pathological processes. Considering its unique role in each of these processes, CS is postulated to be a crucial detrimental mechanism that fosters the development of CVDs [109].

### 5.2. Senescence in Cardiac Pathology

#### 5.2.1. HF

HF emerges from various stressors and underlying factors that lead to the progressive impairment of cardiomyocytes. This impairment leads to the heart’s inability to effectively meet the demands of the body [110]. Multiple research projects have established a correlation between CS and pathways associated with HF pathophysiology [111]. On the one hand, mice with a global deficiency in kinases, which have a protective impact on mitochondria and telomere length, experience advanced HF at the age of six months. These mice exhibit elevated levels of p16, p53, and SA-β-gal, along with a decline in the structure and function of mitochondria within cardiac tissue [112]. In a similar fashion, diabetic cardiomyopathy in mice is characterized by high levels of senescence markers in both cardiomyocytes and CPCs, coupled with impaired mitochondrial function and elevated ROS generation in the heart [113]. On the other hand, the utilization of an angiotensin II receptor blocker and navitoclax has demonstrated the potential to enhance cardiac function and address electrophysiological abnormalities [114]. Furthermore, increased autophagy has been shown to safeguard cardiac function, reducing p16, p21, p53, and several constituents of the SASP in aged mice. Notably, elevated levels of SASP components align with the severity of HF [115].

Collectively, senescence may intersect with several pathophysiological pathways that contribute to HF, such as mitochondrial dysfunction, autophagy, and activation of the neurohumoral system. Nevertheless, whether targeting cell senescence could serve as a viable therapeutic approach for HF remains a subject requiring further investigation.

#### 5.2.2. MI

Acute MI stands as the foremost contributor to global morbidity and mortality. A lot of data strongly imply a connection between CS and the consequences of myocardial ischemia and MI. Generally, MI causes a considerable depletion of cardiomyocytes. Ischemic injury initiates DNA damage, oxidative stress, and disturbances in mitochondrial performance, collectively influencing the predisposition to cardiomyocyte senescence. In a study by Zhu et al., it was suggested that fibroblast senescence, regulated by p53, acts as a barrier against collagen deposition and cardiac fibrosis. This intricate process culminates in cardiac rupture and impaired cardiac dysfunction post-MI [83].

The role of CS in MI pathogenesis remains a topic of debate, as conflicting reports indicate both positive and negative impacts. Notably, mice treated with navitoclax exhibit significantly enhanced survival rates and reduced functional deterioration post-MI. This observation implies that SNCs contribute to unfavorable remodeling. Moreover, standard drug therapies used to mitigate cardiac dysfunction following myocardial infarction (MI) also cause a decline in the presence of markers of CS in cardiac tissue [1]. Conversely, several studies propose a favorable role for cell senescence post-MI. Specifically, the diminished production of SASP components is linked to increased systolic dysfunction and cardiac fibrosis in the post-MI phase. This association suggests that the components of the SASP within the peri-infarct region exert antifibrotic and cardioprotective effects [116]. While a significant amount of data underscores the connection between CS and the repercussions of myocardial ischemia and infarction, much of this evidence is correlational rather than causal.

#### 5.2.3. Hypertrophic Cardiomyopathy

The physiological response of cardiac hypertrophy to biomechanical stress results in an augmentation of the left ventricular mass and a lowering of cardiac compliance [117]. Several pieces of evidence strongly suggest that senescence actively drives the progression of cardiac hypertrophy. To eliminate SNCs in aged mice, Anderson et al. employed a combination of the INK-ATTAC transgenic model and navitoclax treatment. This intervention led to a decrease in cardiomyocyte size and fibrosis, with cardiac function and left ventricular mass remaining relatively stable [26]. From these findings, the researchers reached the conclusion that CS is a factor in age-related cardiac hypertrophy. They also suggested that the removal of SNCs supports cardiomyocyte regeneration, as evidenced by an increase in mononuclear cardiomyocytes positive for proliferation markers Ki67 and EdU [26]. Numerous studies underscore the significance of ROS and mitochondrial dysfunction in instigating age-related senescence in cardiomyocytes [118,119,120,121]. In summary, CS does indeed contribute to age-related cardiac hypertrophy. Nevertheless, the mechanisms governing senescence in cardiomyocytes and their pathological implications warrant further exploration [1,2].

#### 5.2.4. Cancer Therapy-Induced Cardiotoxicity

The intricate molecular mechanisms that underlie cardiotoxicity are constantly under investigation, encompassing facets like mitochondrial dysfunction, oxidative stress, disrupted autophagy, and telomere dysfunction [122]. CS has recently gained recognition as a pivotal contributor to the development of cardiotoxicity resulting from cancer treatments. Cells induced into senescence by anticancer therapies share common attributes with those prompted by other stimuli. Pertinently, eliminating SNCs hold clinical significance as it can ameliorate cardiac systolic dysfunction [26]. In addition, the molecular signatures and patterns exhibited by SNCs, in conjunction with the recognition of specific molecules within blood samples and other biological fluids, hold promise as emerging novel biological markers in the realm of cardio-oncology [74]. Compared to the relatively abundant research examining the role of senescence in doxorubicin-induced cardiotoxicity, there has been less attention given to the function of senescence in cardiac disease induced by radiotherapy. Nevertheless, whether stemming from radiation or chemotherapy, the comprehensive removal of CS can potentially mitigate cardiotoxicity [26].

#### 5.2.5. Cardiac Fibrosis

Cardiac fibrosis is a multifaceted phenomenon distinguished by the excessive generation of extracellular matrix proteins in the myocardial tissue. The proliferation and activation of cardiac fibroblasts mediate this phenomenon [123]. Several investigations have provided evidence linking the presence of senescent fibroblasts to the occurrence of fibrosis [124,125]. However, the nature of the involvement of senescence in fibrogenesis remains uncertain in these investigations. The use of transgenesis or treatment involving navitoclax has demonstrated a reduction in fibrosis, providing evidence for the possible involvement of SNCs in the advancement of cardiac fibrosis. Furthermore, cell cycle arrest brought on by cellular senescence may have the ability to lessen fibroblast proliferation and, in turn, suppress the progression of fibrosis [1].

#### 5.2.6. Diabetic Cardiomyopathy

Cardiac myopathy can arise as a consequence of diabetes mellitus, a specific clinical condition marked by diminished muscle mass, expanded chambers, and compromised ventricular function, all occurring in the absence of CAD [2]. The various elements that play a part in the onset and advancement of diabetic cardiomyopathy encompass a spectrum of elements. These include compromised cardiac insulin metabolic signaling, endoplasmic reticulum stress, elevated oxidative stress, mitochondrial dysfunction, inflammation, and microvascular dysfunction [125]. The diabetic heart exhibits premature telomeric shortening in its CPCs, evident through telomeric shortening and the presence of senescence-associated proteins, namely p53 and p16INK4A. This phenomenon, in turn, escalates the count of senescent myocytes, thus promoting premature myocardial aging and contributing to HF. The suppression of p53 in a mouse model of streptozotocin (STZ)-induced type 1 diabetes (T1DM) prevented cardiac apoptosis during the early phases of diabetes. Moreover, it attenuated cellular senescence induced by diabetes and averted glycolytic and angiogenetic dysfunction. These effects were achieved by enhancing the stability of the hypoxia-inducible factor-1α (HIF-1α) protein and facilitating HIF-1α-mediated genomic transcription [126]. This insight potentially lends theoretical support to diagnosing and treating diabetic cardiomyopathy.

## 6. Therapeutic Approaches

SNCs have been recognized as impactful contributors to aging and age-related CVDs in the last two decades. The supporting evidence for this assertion stems from the identification of SNCs biomarkers in affected tissues, the genetic interventions that disrupt senescence and thereby alter disease pathophysiology, the modification of disease progression by means of transgene-mediated SNC elimination, and the modification of pathological processes through the administration of senolytics. Collaboratively, these advancements strongly indicate that SNCs present a possible target for senotherapy, holding the potential to treat and even prevent CVDs [127].

**Figure 4:**
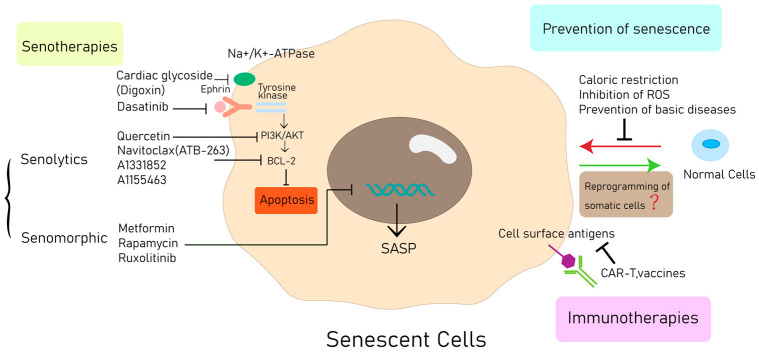
Novel therapeutic approaches for targeting SNCs. PI3K, phosphoinositide 3-kinase; AKT, protein kinase B; BCL-2, B-cell lymphoma 2; SASP, senescence-associated secretory phenotype; ROS, reactive oxygen species.

The therapeutic intervention aimed at SNCs is gaining recognition as a promising and pioneering approach to hindering cardiovascular aging and the advancement of associated diseases. Presently, a range of methods is being utilized to eradicate cardiovascular SNCs in in vitro and in vivo models [128] (Figure 4).

### 6.1. Prevention of Senescence

One potential therapeutic approach involves the prevention of senescence by mitigating pro-senescent stressors. For instance, caloric restriction has demonstrated the ability to effectively inhibit CS by diminishing the activation of the mammalian target of rapamycin (mTOR) signaling pathway via the AMP-activated protein kinase (AMPK) [102,129]. In vitro investigations have indicated that inhibiting the accumulation of ROS, using the ROS scavenger N-acetyl-l-cysteine, can prevent CS in human tissues [130]. In mice with diabetes mellitus or heart failure, the advancement of CS can be decelerated by preventing hypertension and inhibiting the sympathetic nervous system [131]. Furthermore, various methods to inhibit the anthracycline-induced CS of VSMCs and cardiomyocytes have been identified, such as prednisolone and the amplification of LncRNA-MALAT1 [132,133].

### 6.2. Senotherapies

In SNCs, glycolysis is elevated, DNA damage response pathways are heightened, and SASP is present, offering potential avenues for therapeutic intervention [134]. Senotherapeutics constitute a class of medications aimed at eliminating SNCs (senolytics) and mitigating the external effects caused by SNCs (senomorphics or senotatics) [135,136]. Ongoing efforts encompass the screening of repurposed medications and natural products, as well as the development of novel compounds targeting SNCs [11].

#### 6.2.1. Senolytics

SNCs’ anti-apoptotic pathways (SCAPs) have led to the elimination of a significant portion of SNCs that possess pro-apoptotic qualities and are detrimental to tissues, which is achieved through apoptosis. Distinct categories of pro-apoptotic SNCs in humans rely on specific SCAPs for their survival [137]. A variety of techniques have been designed for senolysis, focusing on anti-apoptotic signaling molecules as targets to effectively eliminate SNCs in the context of aging [11]. Dasatinib and quercetin are two examples of agents that promote apoptosis in SNCs by suppressing ephrin receptor-dependent tyrosine kinases and PI3K/mTOR pathways, respectively [138]. Considering their recognized targets, the combination of quercetin and dasatinib has exhibited greater efficacy in eliminating senescence across diverse pathological models. In terms of administration, single or intermittent doses demonstrate considerable advantages compared to continuous senolytic treatment, as they entail fewer side effects [134,139,140,141]. A recent study demonstrated the testing of dasatinib in conjunction with quercetin in clinical scenarios involving dysfunction related to idiopathic pulmonary fibrosis, implying that anti-senescent agents might soon become accessible for human use. Consequently, the optimization of anti-senescent protocols for impending clinical translation holds significant promise.

By targeting the B-cell lymphoma 2 (BCL-2) family of proteins, navitoclax or ABT263 is able to effectively induce apoptosis and eliminate SNCs [142]. This approach has been shown to delay the onset and progression of CVDs such as atherosclerosis, myocardial infarction (MI), ischemia-reperfusion injury, and cardiac aging [26,96,142,143].

A new noteworthy senolytic candidate is digoxin, a cardiac glycoside with a history of application in cardiac disease treatment. The repurposing of established drugs like digoxin holds the potential to expedite clinical translation. In mouse models, the administration of digoxin has demonstrated the capacity to eradicate transplanted SNCs [144,145]. Similar to many other senolytic regimens, intermittent administration of senolytic therapy with digoxin, using a ‘hit-and-run’ approach, has the potential to yield improved clinical outcomes [11].

This avenue of treatment holds substantial promise. After the original hypothesis-driven drug discovery method, many more senolytic compounds have been identified through various methods, including high-throughput screening. Other small molecules that have been found for the targeted elimination of SNCs include specific BCL-xL inhibitors such as A1331852 and A1155463, the flavonol fisetin, piperlongumine, procyanidin C1, a FOXO4-related peptide, and several other compounds [127,146,147,148].

#### 6.2.2. Manipulation of the SASP

SASP inhibitors, often called “senomorphic” drugs, do not have a direct effect on the removal of SNCs. Instead, they function by altering elements of the SNCs secretome that contribute to chronic inflammation and tissue deterioration. Specific drugs among these target the transcription factor nuclear factor kappa B (NF-κB) [149,150], Janus kinases, or STAT pathways. Additional targets encompass the mTOR pathway, p38MAPK and its associated kinases, molecules associated with mitochondrial complexes 1 or 4, heat shock protein 90 (HSP90), and NAD+/NADH metabolism [38]. Several medications that are approved by the Food and Drug Administration (FDA) have been found to possess senomorphic effects. These include metformin, rapamycin, and ruxolitinib [151,152,153,154]. Building on these encouraging findings, initiatives such as the Targeting Aging with Metformin (TAME) trial, supported by the American Federation for Aging Research (AFAR) and others, are in the planning stages. However, the utilization of certain senomorphic drugs poses challenges related to managing potential off-target effects. This includes scenarios where suppressed inflammation might not be beneficial for certain diseases or tissue repair. In contrast to senolytics, which directly eliminate the SNCs responsible for releasing tissue-damaging SASP factors, the need for continuous treatment could be more pronounced with senomorphic drugs [11].

### 6.3. Immune Activation of Target SNCs

Immunotherapies initially designed for cancer treatments, including therapies like chimeric antigen receptor T cell (CAR-T), cytotoxic T lymphocyte (CTL), natural killer (NK) cell, and dendritic cell (DC) therapies, which are currently undergoing clinical applications, have the potential to be repurposed as senolytics. One potential strategy for selectively eliminating SNCs is to target SNC-specific antigens, which are referred to as “seno-antigens”. This approach offers the benefit that tailored treatments can be devised upon identification of a target molecule, even if the complete physiological function of the molecule remains uncertain or effective inhibitors are yet to be found. This approach holds the potential to mitigate off-target effects [11]. For example, a senolytic vaccine aimed at the glycoprotein non-metastatic melanoma protein B (GPNMB) exhibited enhanced physical function in aged mice and extended the lifespan of progeroid mice [155]. Similarly, another senolytic vaccine focused on CD153, identified as a hallmark of obesity-related T cells within adipose tissue, successfully decreased the buildup of senescent T cells [156]. Both vaccine treatments demonstrated effectiveness for several months. Moreover, various antibody–drug conjugates (ADCs) have been identified as having the ability to effectively removing SNCs [157,158].

### 6.4. Bypassing and Reverting Senescence

While CS was initially characterized as an essentially irreversible cell cycle withdrawal, inducing senescence can actually be reversed in vitro, leading to the revival of cell proliferation [159]. Furthermore, a groundbreaking study demonstrated that in vivo reprogramming of somatic cells could reinstate their ability to proliferate [160]. These investigations introduce the intriguing prospect of bypassing senescence therapeutically. Nevertheless, it is essential to exercise prudence with such approaches, as recent studies have indicated that this strategy might heighten the tumor-initiating capacity of cells beyond that of nonsenescent cells [161].

Despite major advances, the translation of senescence-targeting strategies to clinic applications must be undertaken cautiously. This caution arises from the fact that senescence can exert both advantageous and detrimental effects, depending on the specific pathological context [162].

## 7. Conclusions and Outlook

As life expectancy rises, CVDs have become increasingly prevalent. Many prominent pharmacological treatments, designed to address age-related CVDs, have yielded less than satisfactory outcomes, underscoring the necessity for innovative treatment approaches. As a novel research focus in CVDs, emerging evidence suggests the involvement of CS in the pathological progression of CVDs that were triggered by severe acute respiratory syndrome coronavirus 2 (SARS-CoV-2) infection. CS has even been investigated as the key pathogenic mechanism and a promising therapeutic target for SARS-CoV-2 infection. Therefore, in the present post-epidemic era, there is an increasing urgency to elucidate the mechanisms underlying various pathological processes mediated by CS in the human body [163,164].

CS represents a complex cellular state, serving both physiological and pathophysiological functions in processes such as cellular development, the pathogenesis of diseases, and organ dysfunction [165]. Cardiovascular cell senescence is vital in maintaining homeostasis within cardiovascular tissue during tissue regeneration, embryonic development, and wound healing [166]. However, the persistent buildup of SNCs within cardiovascular tissues can impede their function and has been linked to the development of age-related CVDs [167], such as HF, MI, hypertrophic cardiomyopathy, cardiotoxicity, cardiac fibrosis, arrhythmogenic cardiomyopathy, and diabetic cardiomyopathy. Complex molecular pathways intricately govern cardiovascular cell senescence both in vitro and in vivo. Nevertheless, there remains an inadequate grasp of the precise underlying mechanisms that contribute to the dysregulation of cardiovascular cell senescence during the onset of CVDs. At present, there is a deficiency of dependable selective markers to detect senescent cardiovascular cells in vivo [13]. The spatiotemporal identification and noninvasive quantification of individual SNCs in vivo remain challenging [168]. These challenges collectively impede the development of efficacious treatments for CVDs. The successful development of new treatment strategies for cardiovascular aging cells, to alleviate the substantial clinical significance, depends on a thorough understanding of aging biology and the biological specifics and primary cell types that participate in the pathogenesis of CVDs [128]. The selective elimination of SNCs has emerged as a safer approach to senescence during aging [166].

A comprehensive research approach is imperative to target senescent cardiovascular cells accurately, effectively, and safely. This approach should encompass the following aspects: (1) accounting for discrepancies between animal models and human conditions; (2) investigating cellular and model systems representative of natural aging; (3) investigating the mechanism underlying the senescence of cardiovascular cells and its contribution to the initiation and progression of CVDs; (4) identifying distinct spatiotemporal biomarkers and targets specific to senescence in various cardiovascular cell types in vivo; (5) validating the efficacy of established senolytic agents while considering potential side effects; and (6) assessing the potential risk of cancer escalation following the removal of SNCs [128]. With the possibility of therapeutic interventions via targeting, a comprehensive grasp of the manifold roles and mechanisms associated with CS in CVDs not only aids in the development of novel agents, but also facilitates the formulation of appropriate clinical strategies. Nonetheless, it remains imperative to conduct extensive studies for the formulation of precise and accurate treatment strategies for CVDs.

## Figures and Tables

**Table 1:** Senescence initiators, pathology, associated CVDs, and hallmarks of specific cardiac cell types. SASP, senescence-associated secretory phenotype; ROS, reactive oxygen species; HFpEF, heart failure with preserved ejection fraction; SA-β-gal, senescence-associated β-galactosidase; TRF1/2, telomeric repeat binding factor 1/2; γH2AX, phosphorylated histone H2AX; SASP, senescence-associated secretory phenotype; TAF, telomere-associated foci; ATM, ataxia–telangiectasia mutated; Chk2, checkpoint kinase 2; TRF2, TTAGGG repeat binding factor-2.

Cell Type	Initiators	Pathology	Diseases	Hallmarks
Cardiomyocytes	Metabolic dysfunction Telomeric shortening Epigenetic factors SASP	Impaired contractility Abnormal conduction patterns Fibrosis	Heart failure Cardiomyopathy Ischemic heart disease Arrhythmias	SA-β-gal, p16, Telomerase, TRF1/2, p53, p27, γ-H2AX, Telomere length, SASP, TAF
Endothelial cells	Oxidative stress Vascular inflammation Metabolic factors Epigenetic regulation	Vascular dysfunction Fibronectin accumulation Vascular inflammation Impaired vasodilation	Atherosclerosis HFpEF Pulmonary hypertension Peripheral artery disease Ischemic heart disease	SA-β-gal activity, p53, p21, Cell morphology, p16, SASP, γ-H2AX, ATM, Chk2, Telomerase activity
VSMCs	Telomeric shortening DNA damage Oxidative stress Autophagic dysfunction	Inducing local inflammation Impaired smooth muscle Contraction	Atherosclerosis Pulmonary hypertension Hypertension Aortic aneurysm Aortic dissection Myocardial infarction	Cell morphology, SA-β-gal activity, p53, p21, p16, SASP, p27, γ-H2AX, ATM, TRF2, Telomere length,
Fibroblasts	Oxidative stress Hypoxia	Limits collagen expression Prevent excessive fibrosis	Heart failure Cardiomyopathy Arrhythmias	SA-β-gal activity, p53, SASP, p21, p16, p19, γ-H2AXCell morphology
Immune cells	Telomeric shortening Cell debris ROS	Inflammation Impaired cardiac electrical Conduction	Atherosclerosis Heart failure Arrhythmias	Flow cytometry, CD8+CD57+ T cells, CD28-T cells, D4+CD57+ T cells, CD8+CD28- T cells
CPCs	Aging	Fibrotic remodeling Proinflammatory	Ischemic heart diseases	SASP, SA-β-gal, p16, γ-H2AX, Telomere length

## Data Availability

Not applicable.

## Abbreviations

The following abbreviations are used in this manuscript:

**Abbreviation**	**Meaning**
CS	cellular senescence
SASP	senescence-associated secretory phenotype
CVDs	cardiovascular diseases
SIPS	stress-induced premature senescence
RS	replicative senescence
DDR	DNA damage response
hTERT	human telomerase reverse transcriptase
SSBs	single-strand breaks
DSBs	double-strand breaks
ATM	ataxia–telangiectasia mutated
ATR	ataxia–telangiectasia Rad3-related
γH2AX	phosphorylated histone H2AX
CDKN1A	CDK inhibitor p21
cGAS	cyclic GMP–AMP synthase
STING	stimulator of interferon genes
TAFs	telomere-associated foci
mTOR	mammalian target of rapamycin
ROS	reactive oxygen species
OIS	oncogene-induced senescence
mtDNA	mitochondrial DNA
ECM	extracellular membrane
mi-RNA	microRNAs
lncRNA	long non-coding RNAs
EndoG	endonuclease G
SA-β-gal	senescence-associated β-galactosidase
SBB	Sudan Black B
CCFs	cytoplasmic chromatin fragments
p38MAPK	p38 mitogen-activated protein kinase
SAHF	senescence-associated heterochromatin foci
SADs	senescence-associated distention of satellites
H_2_O_2_	hydrogen peroxide
BrdU	bromodeoxyuridine
EdU	ethynyl deoxyuridine
VSMCs	vascular smooth muscle cells
DMD	Duchenne muscular dystrophy
LMNA	lamin A
ET-1	endothelin-1
HF	heart failure
HFpEF	heart failure with preserved ejection fraction
MMPs	matrix metalloproteinases
MI	myocardial infarction
CAD	coronary artery disease
T1DM	type 1 diabetes
STZ	streptozotocin
HIF-1α	hypoxia-inducible factor-1α
SNCs	senescent cells
AMPK	AMP-activated protein kinase
SCAPs	senescent cell anti-apoptotic pathways
BCL-2	B-cell lymphoma 2
NF-κB	nuclear factor kappa B
HSP90	heat shock protein 90
FDA	Food and Drug Administration
TAME	Targeting Aging with Metformin
AFAR	American Federation for Aging Research
CAR-T	chimeric antigen receptor T cell
CTL	cytotoxic T lymphocyte
NK	natural killer
DC	dendritic cell
GPNMB	glycoprotein non-metastatic melanoma protein B
ADCs	antibody–drug conjugates
TGF-β	transforming growth factor-β
SMAD	small mother against decapentaplegic
AMPK	adenosine 5‘-monophosphate (AMP)-activated protein kinase
Rb	retinoblastoma protein
MiDAS	mitochondrial dysfunction-associated senescence
IGF-1	insulin-like growth factor 1
CPCS	cardiac progenitor cells
